# Machine Learning and Deep Learning Algorithms for Skin Cancer Classification from Dermoscopic Images

**DOI:** 10.3390/bioengineering9030097

**Published:** 2022-02-27

**Authors:** Solene Bechelli, Jerome Delhommelle

**Affiliations:** 1 Department of Biomedical Engineering, University of North Dakota, Grand Forks, ND 58202, USA; solene.bechelli@und.edu; 2 MetaSimulation of Nonequilibrium Processes (MSNEP) Group, Tech Accelerator, University of North Dakota, Grand Forks, ND 58202, USA; 3 Department of Chemistry, University of North Dakota, Grand Forks, ND 58202, USA; 4 School of Electrical Engineering and Computer Science, University of North Dakota, Grand Forks, ND 58202, USA

**Keywords:** skin cancer, image classification, deep learning, machine learning, convolutional neural network

## Abstract

We carry out a critical assessment of machine learning and deep learning models for the classification of skin tumors. Machine learning (ML) algorithms tested in this work include logistic regression, linear discriminant analysis, k-nearest neighbors classifier, decision tree classifier and Gaussian naive Bayes, while deep learning (DL) models employed are either based on a custom Convolutional Neural Network model, or leverage transfer learning via the use of pre-trained models (VGG16, Xception and ResNet50). We find that DL models, with accuracies up to 0.88, all outperform ML models. ML models exhibit accuracies below 0.72, which can be increased to up to 0.75 with ensemble learning. To further assess the performance of DL models, we test them on a larger and more imbalanced dataset. Metrics, such as the F-score and accuracy, indicate that, after fine-tuning, pre-trained models perform extremely well for skin tumor classification. This is most notably the case for VGG16, which exhibits an F-score of 0.88 and an accuracy of 0.88 on the smaller database, and metrics of 0.70 and 0.88, respectively, on the larger database.

## 1. Introduction

Skin cancers are among the most frequent types of cancer. For instance, more than 5 million new cases are identified every year in the United States, and it is estimated that one in five individuals will be diagnosed with this disease in their lifetime [1,2]. Having widely available accurate diagnostic tools has thus emerged as a global challenge [3]. Skin cancers are often diagnosed by visual inspection, followed by biopsy and histopathological examination. Given that the 5-year survival rate for melanoma can exceed 90% if detected early [4,5], it is crucial to develop highly automated, reliable, and efficient early diagnostic tools.

Diagnosis methods based on the computational analysis of dermoscopic images of skin lesions have been the focus of recent research [6,7]. This approach had been previously deemed to be challenging and of limited accuracy [8,9,10] because of the wide variety of types of skin cancers and the resulting images [11]. In recent years, work based on machine learning (ML) and deep learning (DL) has led to renewed interest in such diagnostic tools [12,13,14,15,16,17,18,19,20]. Recent studies have employed preprocessing and segmentation to extract geometric information on the skin lesion, e.g., size and shape, to classify skin cancer images [6,21]. As discussed by Pereira et al. [6], an outstanding challenge for this class of methods is the performance of the segmentation step for the identification of the border of the skin lesion. Alternatively, pixel values can be analyzed and allow for the prediction of the nature of the tumor [7,22,23]. Deep learning algorithms [24] have increasingly been used to this end. Convolutional Neural Network (CNN) models have become promising candidates for these applications since they have the potential of capturing the fine-grained variability in skin lesion images [7]. Morphological analyses have recently been performed by the combination of segmentation and pre-processing steps to obtain gray-level skin lesion images with a pre-trained Levenberg–Marquardt neural network for clustering and classification of images from the PH2 database [25]. Transfer learning has also been employed to achieve high-accuracy predictions on skin cancer images from the HAM10000 database using the MobileNet CNN [26]. Recent work on the use of CNN for skin cancer prediction has also pointed to outstanding challenges in the inclusion of the full range of patient population and all types of melanoma subtypes [27]. Furthermore, optimization algorithms for the fine-tuning of hyperparameters are currently under development to improve accuracy [28] and methods to detect shortcut learning in the training of CNN models on the ISIC Archive dataset have been recently proposed [29]. Recent work has also focused on the application of interpretable machine learning methods to the multi-class segmentation and classification of skin legion images [30].

Here, we assess the accuracy of various ML and DL approaches for the development of diagnostic tools on the sole basis of dermoscopic images. More specifically, the aim of this work is to design a diagnostic tool that classifies skin lesion images between two classes (benign and malignant), rather than based on multi-class segmentation and classification tasks [26,30]. Given the impact of the training/testing dataset on the results [27], we systematically test a wide range of ML and DL models on the same dataset, the publicly available Kaggle database, to obtain a consistent set of metrics for their performance. ML models include logistic regression, linear discriminant analysis, k-nearest neighbors classifier, decision tree classifier, and Gaussian naive Bayes, while DL models consist of a custom Convolutional Neural Network (CNN) model and pre-trained models, such as VGG16, Xception, and ResNet50. Our results show the overall increase in performance obtained by using DL methods, custom CNNs or with pre-trained models through transfer learning. We also demonstrate that the implementation of ensemble learning maximizes performance, as shown by improvements in the set of metrics (accuracy, precision, recall, and F-score). Finally, we quantify the impact of imbalance in the training dataset on the performance of the DL models.

The paper is organized as follows. We start by presenting the datasets, machine learning, and deep learning models, as well as the ensemble learning approaches used in this work. We analyze the performance of each of these models on a balanced database (Kaggle database) using metrics that quantify the accuracy of the model and analyze the fraction of false negatives obtained in each case. We then extend our analysis of the performance of deep learning models to the larger and more imbalanced HAM10000 database. We finally draw the main conclusions from this work in the last section.

## 2. Materials and Methods

### 2.1. Data

**Figure 1:**
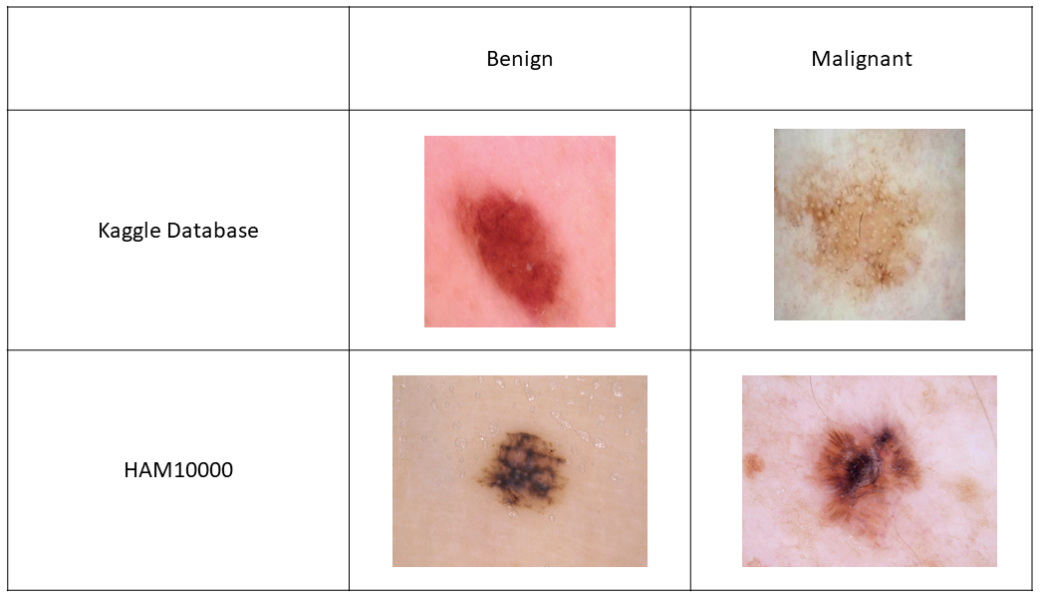
Example of images taken from the Kaggle and HAM10000 databases for classification of benign vs. malignant tumors.

While several datasets of dermoscopic images are available, predicting the malignancy of skin lesions is a complex task that requires large amounts of data to parameterize and train accurate models. As a result, few datasets actually contain a sufficiently large number of images to train machine learning (ML) and deep learning (DL) models for skin lesion classification [31]. In this work, we use two publicly available datasets. The first dataset is provided by the Kaggle community and comes from the Archive of ISIC [32]. It contains a total of 3297 dermoscopic images divided into two classes, “benign” and “malignant”. This dataset is evenly balanced between the two classes and therefore allows for an unbiased study of skin cancer classification via ML and DL. This is unusual, as hospital databases are often imbalanced since their primary objective is to identify malignant tumors [33]. To our knowledge, while many notebooks (>100) are available on Kaggle, there is no ongoing challenge for the classification of benign vs. malignant skin lesions. The second dataset is taken from the Harvard Dataverse (HAM10000) [34], and contains a total of 7 different types of lesions, with lesions belonging to the “benign” class listed as benign keratosis-like lesions (bkl), dermatofibroma (df), melanocytic nevi (nv), vascular lesions (vasc), and lesions belonging to the “malignant” class listed as Actinic keratoses (ak), intraepithelial carcinoma/Bowen’s diseases: bow, basal cell carcinoma (bcc) and melanoma (mel). This dataset contains over 10,000 images, but is highly imbalanced with a large fraction of the dermoscopic images belonging to the “benign” class. While there is a challenge based on the identification of the seven types of skin lesions, there is, to our knowledge, no ongoing challenge on the classification task, benign vs. malignant, for the HAM10000 database. Examples of images taken from each database are shown in Figure 1.

### 2.2. Machine Learning Models

We examine the performance of several supervised ML algorithms, including Logistic Regression (LR), Linear Discriminant Analysis (LDA), k-nearest Neighbors classifier (KNN), Decision Tree Classifier (CART), and Gaussian Naive Bayes (GNB). We choose these algorithms for the following reasons. The first method (LR) is especially suited for the classification of binary problems and has been often used in biomedical applications [35]. LR is a parametric method that calculates the class membership probability for one of the two categories in the dataset. The second algorithm (LDA) is commonly used for supervised pattern classification and produces linear decision boundaries between classes [36]. The next approach (KNN) relies on proximity. This method uses a metric that assigns to each item a specific category depending on its closeness to similar data points, and its versatility makes it an appropriate choice to study classification problems [35]. Decision tree classifiers, such as the decision-tree procedure called Classification And Regression Trees (CART) [37], have been used increasingly in biomedical applications [38]. We also employ Gaussian Naive Bayes (GNB) methods, which originate from Bayes’ theorem and are among the most efficient classifiers [39]. In addition to using a single ML technique to classify images, we leverage an ensemble learning approach to maximize accuracy [40,41,42,43]. For this purpose, we consider groups of ML algorithms and average predictions from each of the ML models in the group with a soft-voting decision. We define three different ensembles or groups of ML methods, noted as E1, E2, and E3. E1 is a classifier that maximizes diversity and averages predictions from very different ML methods (LR, KNN, and GNB). E2 averages predictions from all the ML models used in this work (LR, LDA, KNN, CART, and GNB). Finally, E3 gathers the predictions from the three ML methods (LR, LDA, and CART) that exhibit the best performance.

### 2.3. Deep Learning Models

#### 2.3.1. Custom CNN Model

**Figure 2:**
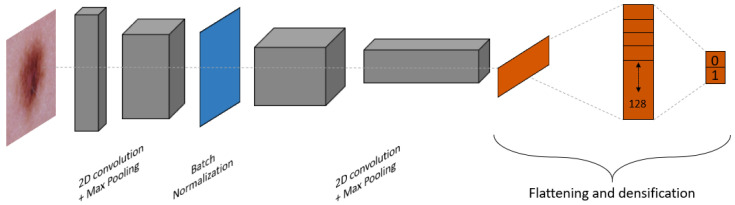
Schematic representation of the layout for the custom CNN model.

**Figure 3:**
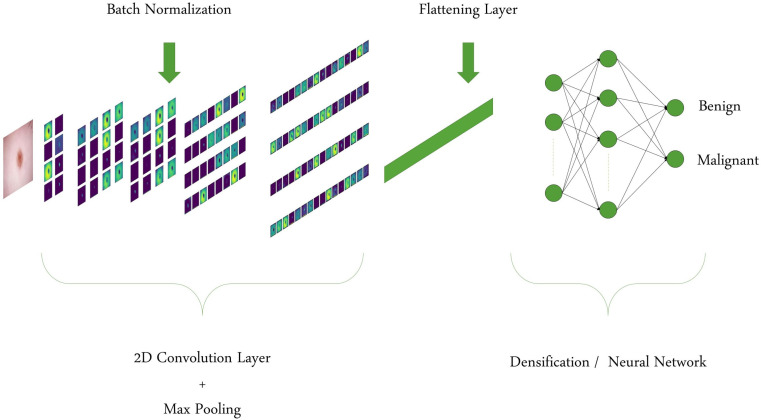
Visualization of how an example of a skin lesion image is processed by the custom CNN model is shown layer by layer.

The first Deep Learning (DL) model we consider is a custom Convolutional Neural Network (CNN) model. This type of model is commonly used in image classification thanks to the performance of convolution processes on shape extraction. The custom model is composed of a total of 11 layers. The characteristics of the layers for the custom CNN are summarized in Table 1. A schematic representation of the layout for the custom CNN is provided in Figure 2, and an example of how it processes an image of skin lesion is provided in Figure 3.

#### 2.3.2. Transfer Learning

Since a complete training of a new deep learning model can be computationally expensive, we also examine the performance of transfer learning approaches [44]. We consider three different pre-trained models: Xception [45], VGG16 [46] and ResNet50 [47]. Xception relies on a CNN approach [45]. Unlike Inception which allows for correlation between channels, i.e., color and spatial changes, Xception assumes that different channels are independent of each other and learn separately [48]. VGG16 is also a CNN model [46], which was initially developed to study the impact of the network depth when using small convolution filters. ResNet50 is a residual neural network. Its architecture is derived from VGG16, with shortcuts added to provide a residual version of the network [47]. All three models were trained on the ImageNet database, which contains over a million images. Pre-trained models recently received increased attention for cancer prediction [49,50,51,52,53]. These models can potentially decrease training time. However, their success ultimately hinges on the similarity between the type of images on which the hyperparameters were fine-tuned and the classification task at hand. To assess this, we split the classification process into two steps. In the first step, the base models for Xception, VGG16, and ResNet50 are embedded in a neural network. The base layers are frozen, and the weights of the outer layers (embedding neural network) are optimized. In the second step, several base layers are retrained to improve accuracy, and both the weights of the retrained base layers and of the outer layers (embedding neural network) are optimized.

### 2.4. Technical Details and Evaluation Metrics

For each system, we split the data between a training dataset (80%) and a testing dataset (20%). In addition, we use a validation dataset for the DL models. This validation dataset is taken out of the training set and consists of 20% of the data. The dataset can be described as discrete since there are two distinct classes, benign and malignant, to which we attribute the values of 0 and 1, respectively. The results are 10-fold cross-validated and the dataset is shuffled so as to avoid any bias or misrepresentation. Feature engineering is carried out as follows. The classification models are based on pixel values. Images are 224×224 pixels and pixel values are rescaled between 0 and 1 and vectorized. In addition, for DL models, we used data augmentation techniques (random rotations, random zooms, and random flips) to mimic the range of conditions (magnification, light, orientation) under which dermoscopic images are obtained [54,55,56]. We build the ML and DL models using the Keras module from TensorFlow (version 2.6). The computations were run on a compute node of two 18-core Intel Xeon Gold 6140 processors, with 192GB of RAM per node.

We use four different evaluation metrics to assess the relative performance of each of the algorithms. The first metric we use is accuracy (*a*), defined as the ratio of correct predictions to the total number of predictions. Distributing the predictions among four classes, with the two types of correct predictions, TP (True Positive) and TN (True Negative), and the two types of incorrect predictions, FP (False Positive) and FN (False Negative), we can write the accuracy as

(1) $$a=\frac{\mathrm{TP}\;+\;\mathrm{TN}}{\mathrm{TP}\;+\;\mathrm{TN}\;+\;\mathrm{FP}\;+\;\mathrm{FN}}$$

Accuracy provides a simple measure of how good a model is at classifying the images. However, this metric lacks sensitivity, especially for imbalanced datasets. To refine the analysis of the relative performance of the models, we use two additional measures, known as precision and recall. The precision *p* is given by

(2) $$p=\frac{\mathrm{TP}}{\mathrm{TP}\;+\;\mathrm{FP}}$$

Precision provides the ratio of correct “positive” predictions to the total number (correct or incorrect) of “positive” predictions. The higher the precision, the better the model is at distinguishing between benign and malignant tumors. The recall *r* is calculated as follows:

(3) $$r=\frac{\mathrm{TP}}{\mathrm{TP}\;+\;\mathrm{FN}}$$

Recall gives the ratio of correct “positive” predictions to the actual number (correct or incorrect) of “positive” cases. The fourth evaluation metric we use is the F-score. It is defined as the harmonic mean of the precision and the recall:

(4) $$\mathrm{F-score}=2\frac{p\times r}{p+r}$$

The F-score is especially meaningful for an imbalanced dataset like HAM10000. We focus on these 4 metrics, rather than on accuracy only, in our discussion. Indeed, recall and F-score take into account false positives and false negatives, which are particularly significant for cancer diagnosis applications.

We also assess the diagnostic ability of the classifiers by determining the receiver operating characteristic (ROC) curve. The ROC curve is obtained by varying the threshold and plotting the true positive rate (TPR) as a function of the false positive rate (FPR). The TPR is equal to the recall *r*, while FPR is given by

(5) $$\mathrm{FPR}=\frac{\mathrm{FP}}{\mathrm{FP}\;+\;\mathrm{TN}}$$

We then calculate the area under the ROC curve (AUC) for each model to provide a comparison between the models. When ensemble learning is used, the TPR, FPR and ROC curve are calculated by averaging the probabilities obtained for each of the models.

## 3. Results and Discussion

### 3.1. Kaggle Database

#### 3.1.1. ML Models

**Figure 4:**
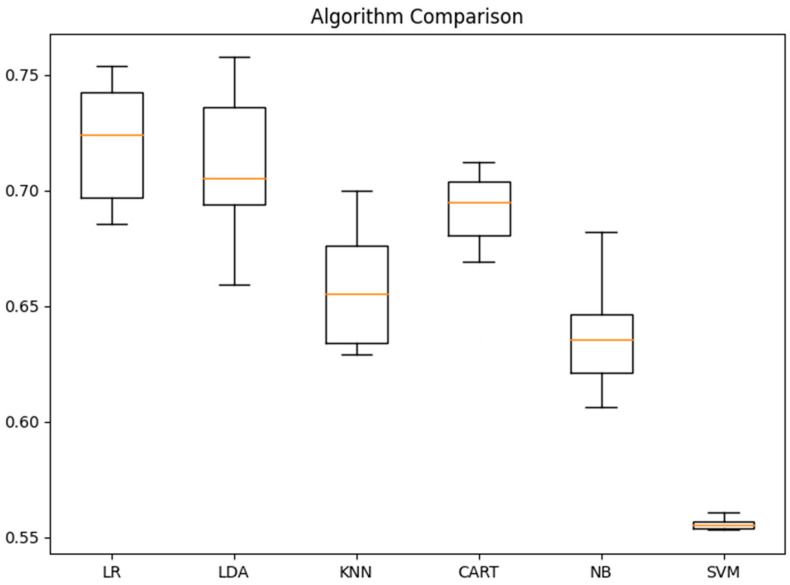
Accuracy for each of the ML models as applied to the dermoscopic images from the Kaggle dataset.

We start by discussing the accuracy of the ML models for the image classification of cancerous skin tumors (see Figure 4). In order of increasing accuracy, we find an accuracy of 63.7%, 65.8%, 68.9%, 71.1%, and 72.1% for GNB, KNN, CART, LDA, and LR, respectively. These results show that linear models tend to provide better performance for the binary task (malignant/benign) involved in skin cancer classification. This, in turn, implies that the features of skin cancer images can be broadly separated into two classes by a straight line. However, the lower performance of the LDA algorithm points to a non-normal distribution of these features, which would not fulfill the assumption required by the LDA model to accurately perform the classification task [57]. Although the accuracy of a model is, as a general rule, a very good indicator of its performance, medical applications require as few false negatives as possible and, as such, the F-score, as well as other metrics, also need to be examined carefully. We summarize in Table 2 the accuracy, F-score, precision, and recall for each ML model.

**Figure 5:**
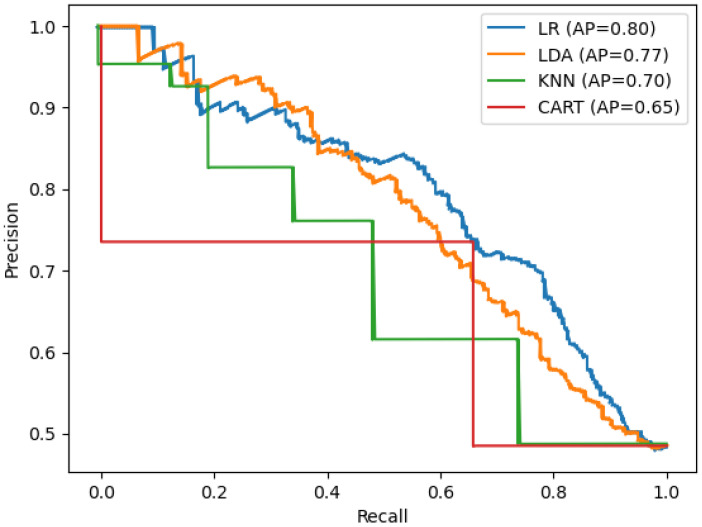
Precision-Recall curve for the 4 highest-performing ML models for skin cancer prediction.

Overall, all ML models are found to exhibit a significant number of false negatives, as shown by the lower values obtained for the recall (all below 0.68) than for the precision, all above 0.73. This is also exhibited by the relatively low values obtained for the F-score, which are all below 0.35. To analyze further the results, we examine the precision-recall curves obtained in this work (see Figure 5). We also evaluate the area under the precision-recall curve for each model. This provides a measure of the sensitivity and performance of the model. The highest area under the curve is obtained for the LR model in line with the results observed for accuracy. Looking at low recalls levels in Figure 5, we find that, for the LR model, the precision remains of 1 for a higher value of *r* (above 0.1) than for the LDA model (only for *r* up to 0.05). Furthermore, LR and LDA are the only models that exhibit the maximum precision performance of 100%. Indeed, the other ML models exhibit a maximum precision either close to 95% (KNN) model and under 80% for all other models, even for very low *r*.

We now turn to the results obtained with ensemble learning. Table 3 shows the metrics obtained for the three ensembles introduced in Section 2.2. Ensemble learning improves significantly several metrics while maintaining the same accuracy as the highest performing ML model. This is most notably the case for the F-score and recall, as shown by a comparison with the metrics obtained for individual ML models. For instance, CART exhibited the highest F-score (0.35) for an individual ML model, and the F-scores for ensemble models are all above 0.62. Similarly, for the recall, the highest performing individual ML model was LR, with a recall of 0.6, while ensemble models are all above 0.79.

**Figure 6:**
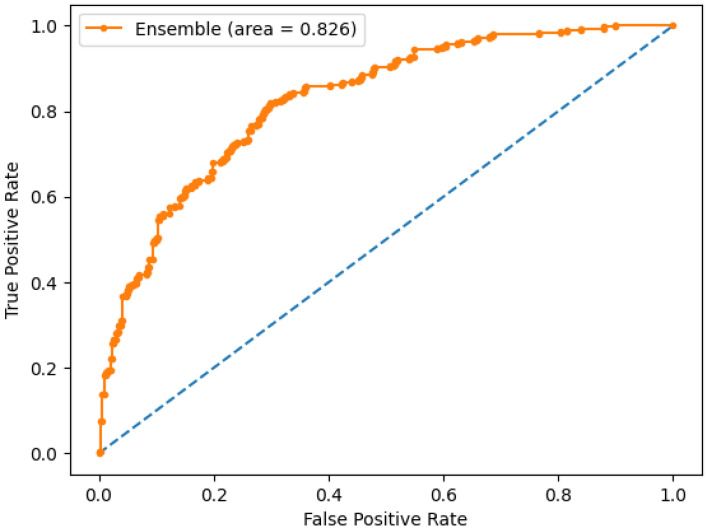
ROC curve for ensemble model E2.

We also compare the ROC curve, as well as the area below the ROC curve (AUC), for each of the ensembles. The ROC curves are similar for all three ensembles, with the maximum AUC obtained for ensemble E2. We show in Figure 6 the ROC curve for ensemble E2, and find areas under the curve of 0.81, 0.83, and 0.81 for ensembles E1, E2, and E3, respectively. This confirms that ensemble models built on linear ML models provide the optimal ML approach for skin cancer prediction models.

#### 3.1.2. Deep Learning Models

**Figure 7:**
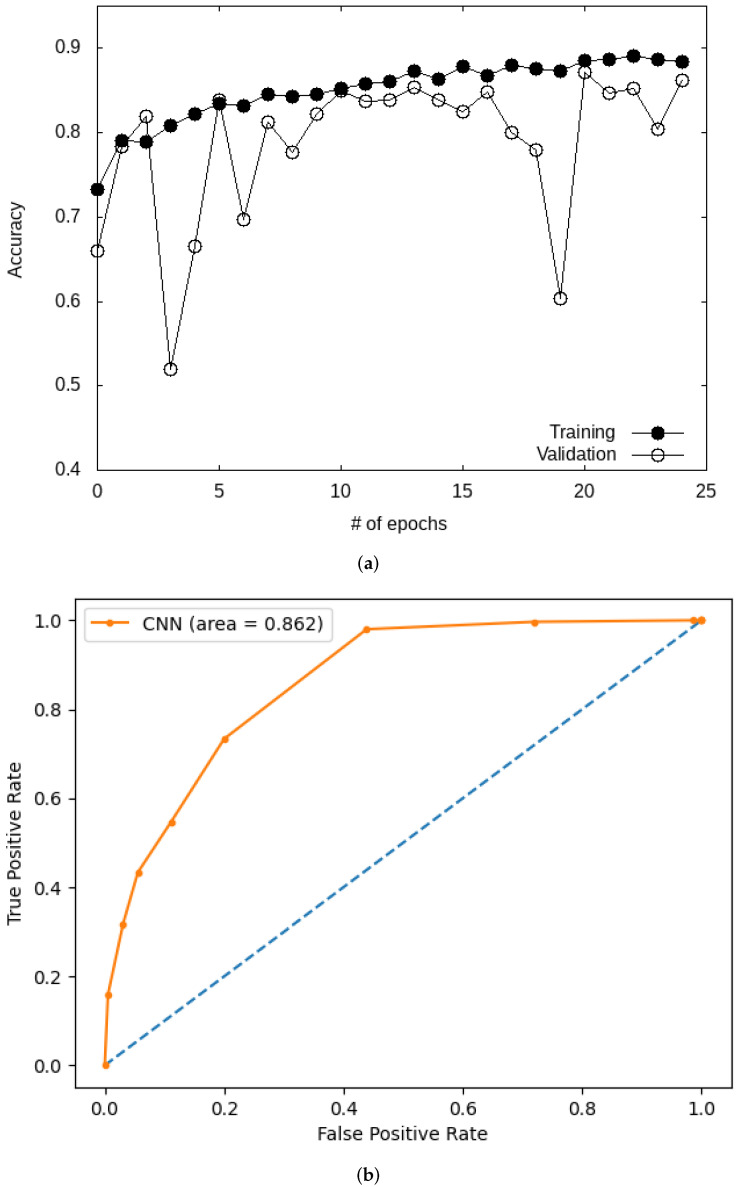
Accuracy (**a**) and ROC (**b**) curves for the custom CNN model on the Kaggle database.

We start by commenting on the results obtained with the custom CNN model. We plot in Figure 7 the variation of the accuracy for the CNN during training. Figure 7 shows an increase in accuracy with the number of epochs for both the training and the validation datasets. The final value reported for the accuracy is 0.83, while other metrics give an F-score, precision, and recall of 0.84, 0.94, and 0.76, respectively (see Table 4). The custom CNN model is found to outperform ML models. The performance of the model is further studied by examining the ROC curve (Figure 7), with a value obtained for the AUC of 0.86. This value is of the same order as those found in previous reports of AUC of about 0.71 obtained on larger datasets [58].

**Figure 8:**
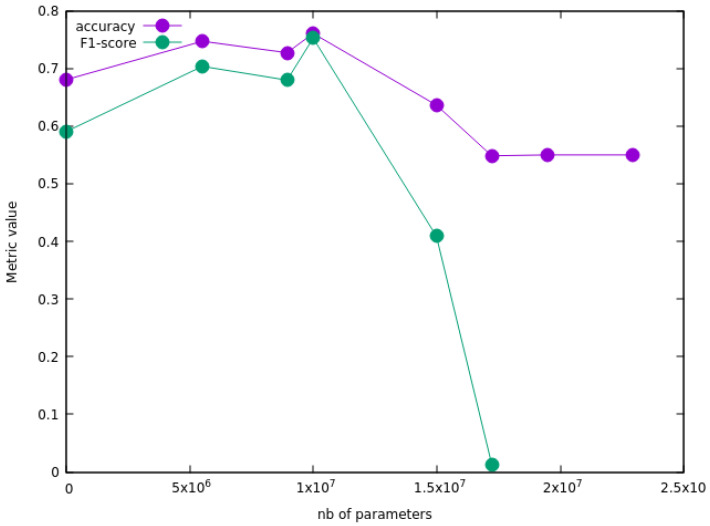
Accuracy and F-score for ResNet50 as a function of the number of base hyperparameters that are unfrozen and re-trained during the “Fine-tuning” step.

We now turn to the results obtained using pre-trained models. A baseline for the performance of these models can be obtained by carrying out a “Frozen base training”, i.e., by taking the base hyperparameters as they were initially fine-tuned and training the embedding neural network only. The baseline performance so obtained is shown in Table 4, with an accuracy ranging from 0.71 (Xception) to 0.85 (ResNet50) and a F-score ranging from 0.71 (Xception) to 0.85 (ResNet50). Interestingly, ResNet50 performs well without any re-training of the base hyperparameters. The next step consists in unfreezing some of the base layers and re-training the corresponding hyperparameters to achieve maximum performance. This second stage is termed “Fine-tuning training” and requires a systematic study of the impact of fine-tuning the hyperparameters on the metrics. We show in Figure 8 the change in accuracy and F-score as a function of the number of re-trained base parameters during the “Fine-tuning training” step of the ResNet50 model. This plot shows that the performance can be optimized by re-training part of the base layers, and leads to the optimal performance metrics reported in the bottom half of Table 4.

**Figure 9:**
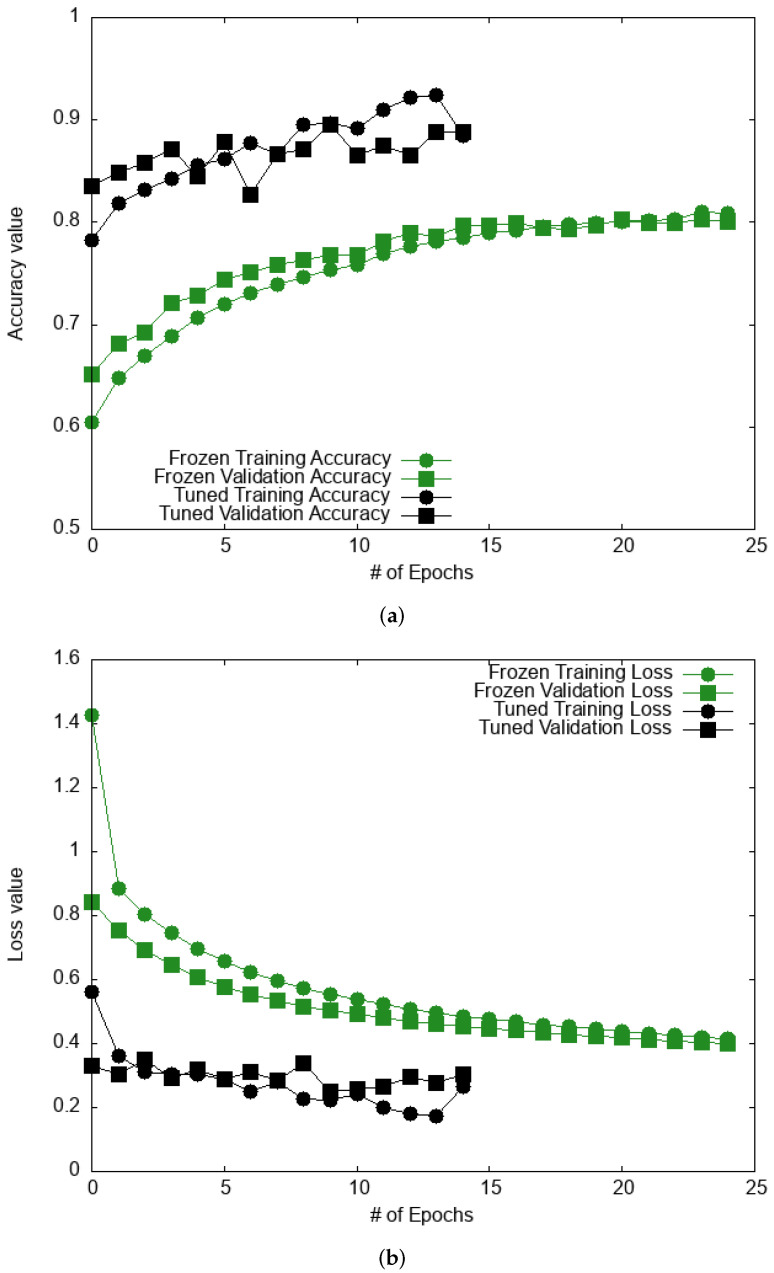

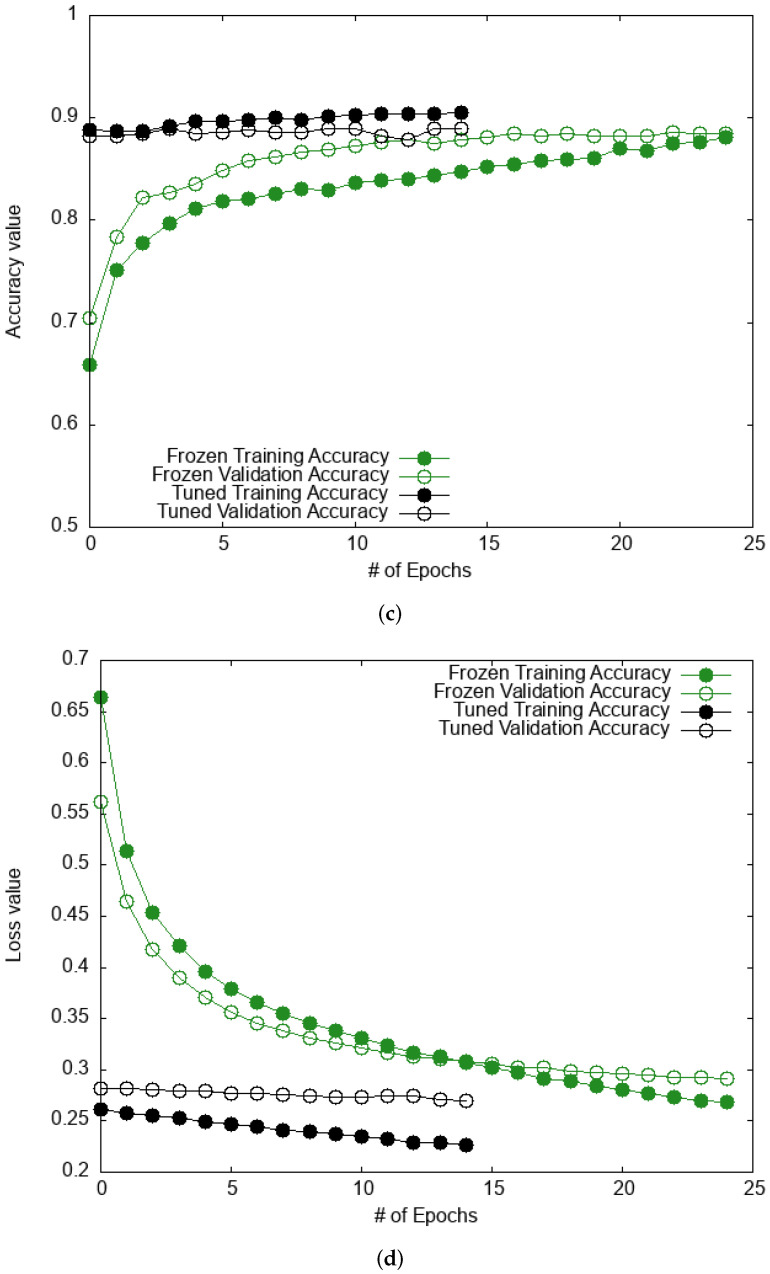
Accuracy and loss evaluation as a function of the number of epochs for VGG16 in (**a**,**b**), respectively, and for ResNet50 in (**c**,**d**), respectively, on the Kaggle database. Results are shown for both the “Frozen base” and “Fine-tuning” steps.

We show in Figure 9 plots for the accuracy and loss during training for the two most accurate models, VGG16 and ResNet50. Figure 9 establishes that both models reach a plateau for both accuracy and loss after only a few epochs without exhibiting any overfitting. These final results exhibit a marked improvement in performance, with ResNet50 and VGG16 displaying similar performance. ResNet50 yields 0.87 in accuracy and 0.87 for the F-score, while Xception exhibits a lower accuracy (0.80) and F-score (0.82). The results are found to be an improvement over prior DL studies on the ISIC dataset [4], which reported a value for the accuracy of 0.76. The results are also in line with recent results obtained with a ResNet50 model [59] on the MClass-D dataset [60]. All three pre-trained models exhibit a greater accuracy and F-score than the ML models discussed in the previous section. We also add that the CPU cost for fine-tuning pre-trained models is roughly an order of magnitude greater than that necessary to train the custom CNN model. Indeed, the custom CNN model requires about 4 min (CPU time) to be trained, when compared to about 40 min for VGG16.

### 3.2. HAM10000 Database

**Figure 10:**
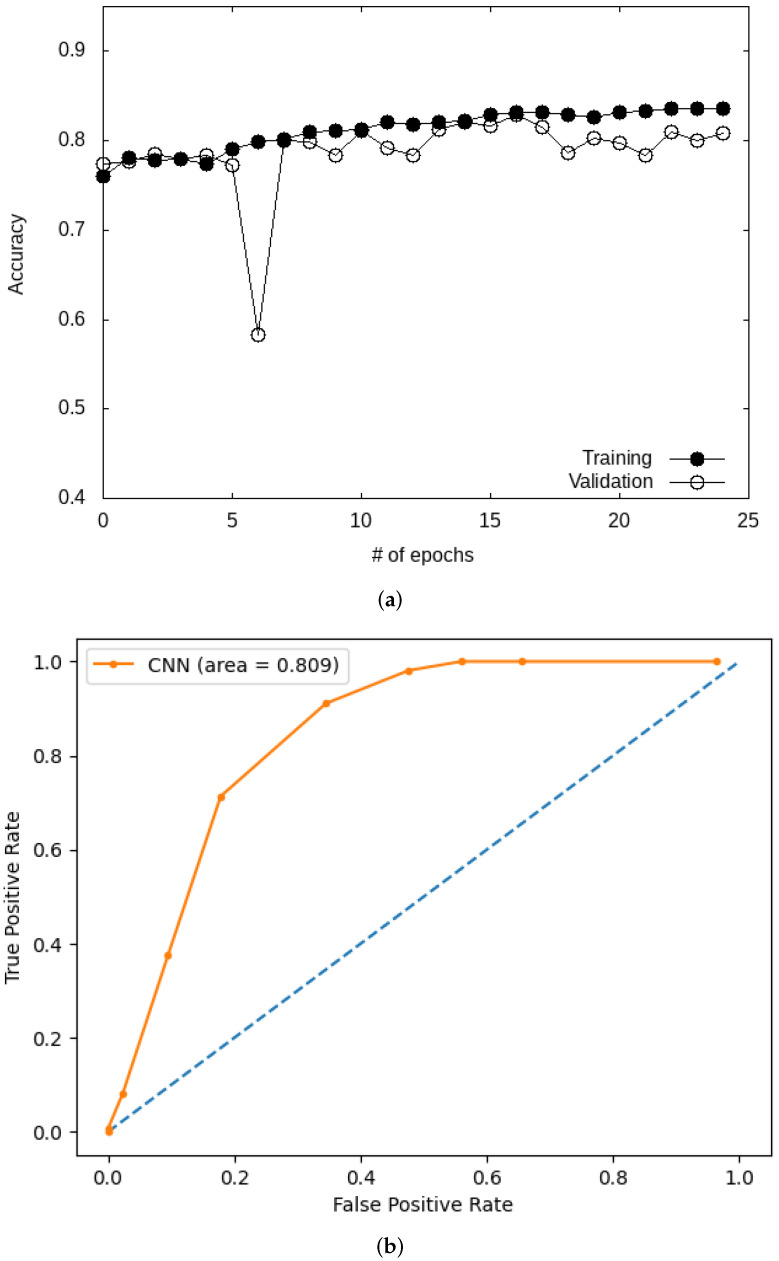
Accuracy (**a**) and ROC curve (**b**) for the custom CNN model (HAM10000 database).

Given the better performance of DL models when compared to ML models for the Kaggle database, we focus in this section on applying DL models to the HAM10000 database. We start with the results obtained for the custom CNN model. Figure 10 shows how accuracy varies for training and validation as a function of the number of epochs. The results show that the accuracy reaches a plateau after about 20 epochs, with an AUC of 0.81 (see in Figure 10b). All four metrics obtained with this model are given in Table 5, and exhibit a decrease, most notably for the F-score, for the HAM10000 database in comparison with the Kaggle database. This likely results from the imbalanced nature of the HAM10000 database.

**Figure 11:**
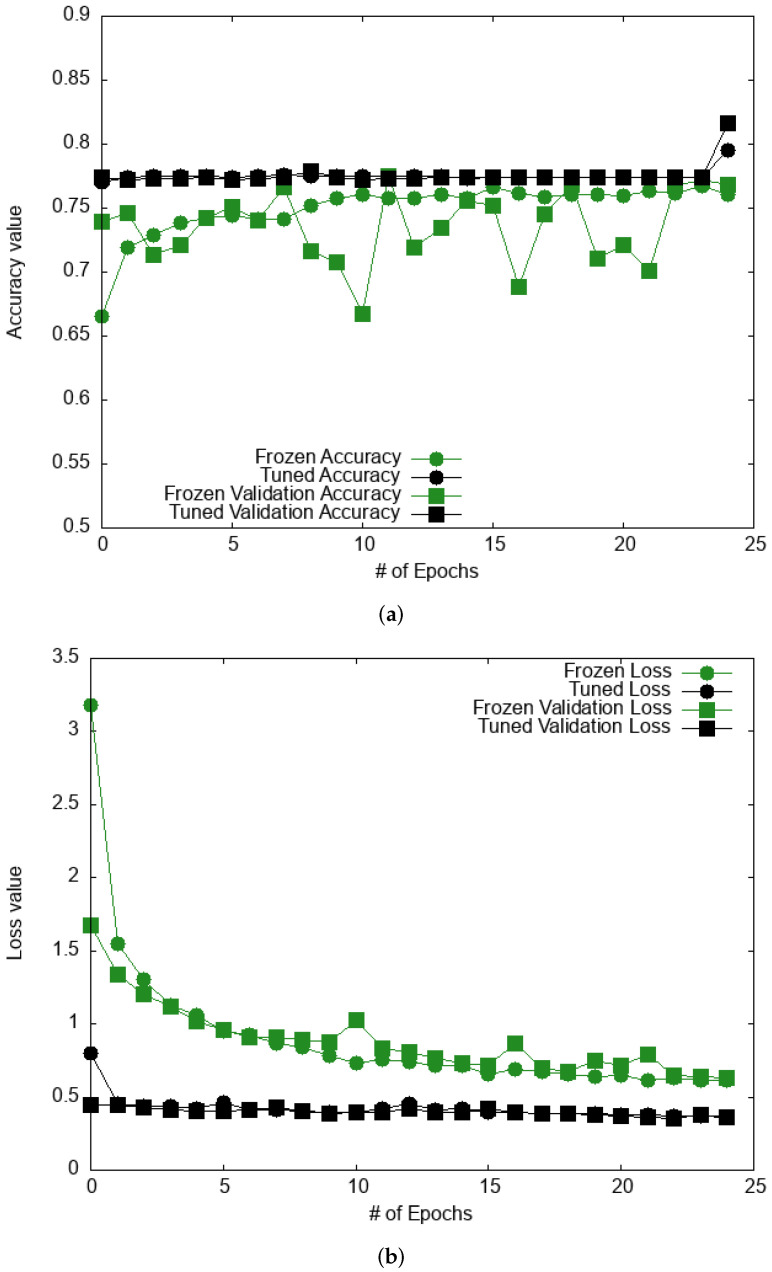

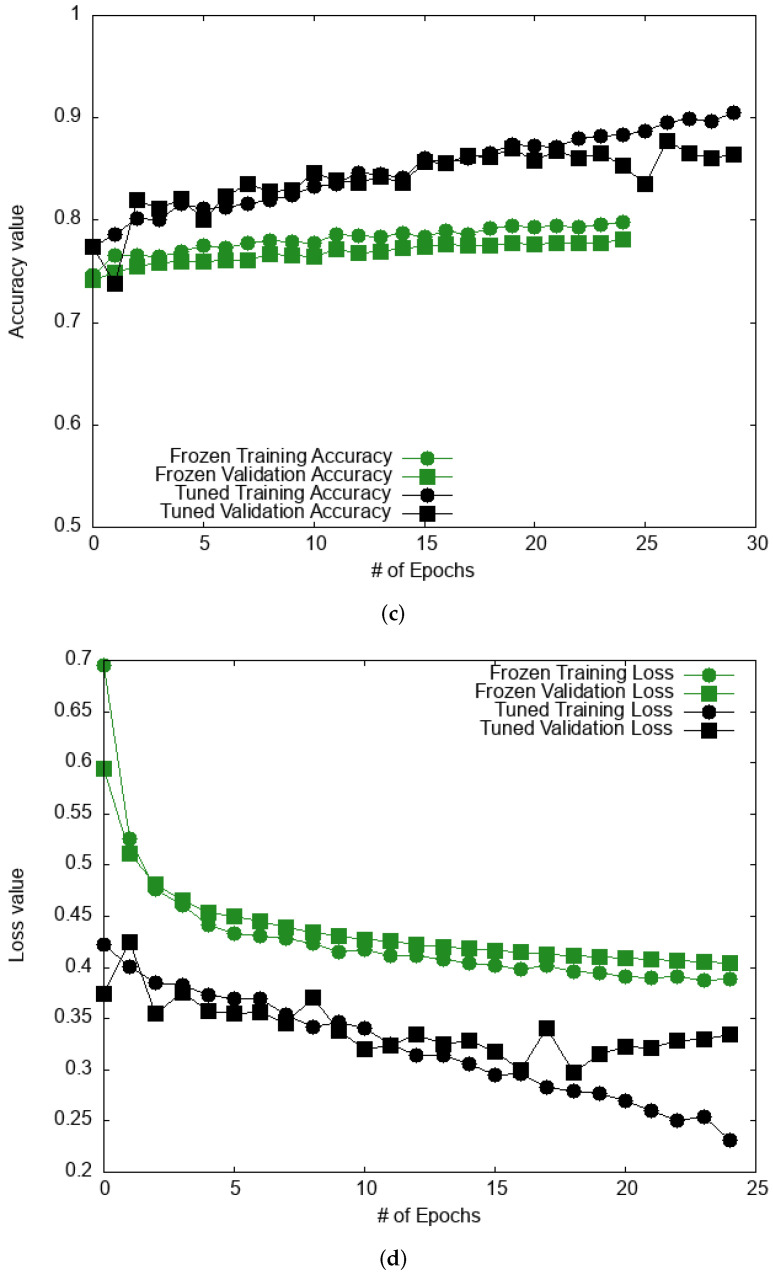
Accuracy and loss evaluation through the number of epochs for the Xception pre-trained model in (**a**,**b**), respectively, and for the VGG16 pre-trained model in (**c**,**d**), respectively, during the two training stages (“Frozen base training” and “Fine-tuning training”).

We now examine the accuracy of pre-trained models (see Figure 11). We follow the same protocol as for the Kaggle database, with for each pre-trained model. The first stage of training involves freezing the base layers and training the embedding neural network. The second stage consists of the partial unfreezing of the base layers with a systematic search for the optimal number of unfrozen layers and re-trained parameters. Very early during the second stage, the accuracy for the Xception model remains constant (about 0.84) and does not show any increase with the number of epochs. The loss function (see Figure 11) reaches a minimum after a few epochs and remains constant afterward, showing that the model is not learning any longer. On the other hand, the accuracy for both VGG16 (also shown in Figure 11) and ResNet50 increase with the number of epochs, leading to a converged accuracy of 0.88 and 0.87, respectively. The other metrics (precision, recall, and F-score) are also provided in Table 5. We obtain the highest precision for VGG16 with a value of 0.68, followed by ResNet50 and the Xception with 0.51 and 0.43. The highest recall is obtained for ResNet50 followed by VGG16 and Xception with values of 0.76, 0.71, and, 0.61 respectively. Lastly, we find the highest F-score for VGG16 with a value of 0.69 compared to ResNet50 (0.61) and Xception (0.50). These results show that VGG16 and ResNet50 exhibit a similar performance on this database in terms of accuracy and F-score with an overall better precision for VGG16. This makes VGG16 the most efficient model overall for skin cancer detection.

We compare these results to those obtained with ensemble learning on pre-trained models (see Table 5) by averaging the predictions of all three models with a hard voting decision. Although the ensemble accuracy is slightly lower than that observed for VGG16 (0.86 vs. 0.87), the ensemble precision improves and reaches 0.79, while the F-score remains the same at 0.70. This shows that precision can be improved without losing performance on the accuracy and F-score. We add that, in this work, we used pre-trained models and CNN models to directly classify the types of tumors. Pre-trained models may also be used as resource extractors, before another ML classifier is applied. As shown by Rodrigues et al. [50], the combination of a DenseNet201 extraction model with a KNN classifier can lead to very high accuracy (between 0.93 and 0.97 depending on the database). This requires a systematic study however since, for instance, our work shows better performance of the ResNet50 model than in previous work [50]. Previous studies have also focused on improving data preprocessing through data augmentation and the application of kernels and filters to remove noise and artifacts [49]. While such protocols tend to improve results for ResNet50 [49], we find that our work led to better performance for VGG16, as shown by the greater accuracy reported in this work. Finally, our results reveal that our approach leads to higher precision and a similar F-score for Xception, when compared to those found in previous work [53].

## 4. Conclusions

In this work, we compare the relative performance of ML and DL models for the analysis of dermoscopic images of skin lesions. While the analysis of such images had long been thought to be challenging and of limited accuracy [8,9,10], recent ML and DL studies have drawn considerable interest and shown tremendous promise [7,12,13,14,15,16,17,18,19,20]. Here, we focus on developing ML and DL models to identify whether a tumor is malignant or benign on the sole basis of dermoscopic images. Among ML models, linear approaches (linear regression and linear discriminant analysis) are found to exhibit the highest accuracy. Our work also shows that DL models are generally found to outperform ML models, most notably when convolutional neural networks (CNN) models are employed to capture the fine-grained variability of dermoscopic images. We analyze the performance of CNN models, either specifically built and trained for this task, or pre-trained, and partially re-trained, models including ResNet50, Xception, and VGG16. Our results show that, in the absence of any re-training, ResNet50 provides a very good overall performance when accuracy, precision, recall, and F-score are taken into account, and thus constitutes an excellent basis if additional methods, either at the pre-processing stage or when resources and features are extracted, are added upon the model. Furthermore, upon partial re-training, VGG16 exhibits the best overall performance. Finally, our results provide a measure for the significance of the nature, and balance, of the training dataset, with much greater performance being achieved when a balanced dataset is used.

## Figures and Tables

**Table 1:** Summary of our custom model layers by layers with their specificities.

Layers	Specificities
2D Convolutional layer	# of filters = 8, size = 3
Max pooling	
2D Convolutional layer	# of filters = 16, size = 3
Max pooling	
Batch normalization	
2D convolutional layer	# of filters = 32, size = 3
Max pooling	
2D Convolutional layer	# of filters = 64, size = 3
Max pooling	
Flattening layer	
Densification	128
Densification	2 (number of classes)

**Table 2:** Metrics for each of the ML models for skin cancer prediction using the Kaggle database.

Model	Accuracy	F-Score	Precision	Recall
LR	0.72 (0.02)	0.34	0.8	0.60
LDA	0.71 (0.03)	0.33	0.75	0.59
KNN	0.66 (0.02)	0.24	0.83	0.34
CART	0.69 (0.02)	0.35	0.73	0.68
GNB	0.64 (0.02)	0.28	0.76	0.49

**Table 3:** Metrics for skin cancer prediction using Ensemble Learning.

Ensemble	Models	Accuracy	F-Score	Precision	Recall	AUC
E1	LR, KNN, GNB	0.71	0.62	0.49	0.83	0.81
E2	LR, LDA, KNN, CART, GNB	0.73	0.66	0.55	0.83	0.83
E3	LR, LDA, CART	0.72	0.66	0.57	0.79	0.81

**Table 4:** Performance of DL models on the Kaggle database. The first line lists the metrics for the results obtained with the custom CNN model. The next lines list results obtained with pre-trained models, when the base hyperparameters are kept the same as in the original model and only the weights for the embedding neural network are optimized (Frozen base), and when some of the base layers are trained (Fine-tuning).

Phase	Model	Accuracy	Precision	Recall	F-Score
Complete training	Custom CNN	0.84	0.94	0.76	0.84
	Xception	0.71	0.65	0.78	0.71
Frozen base	VGG16	0.81	0.77	0.82	0.80
	ResNet50	0.85	0.81	0.88	0.85
	Xception	0.80	0.97	0.71	0.82
Fine-tuning	VGG16	0.88	0.93	0.83	0.88
	ResNet50	0.87	0.89	0.84	0.87

**Table 5:** Performance of DL models (custom CNN and pre-trained) for the HAM10000 database.

Phase	Model	Accuracy	Precision	Recall	F-Score
Complete training	Custom CNN	0.82	0.50	0.44	0.47
	Xception	0.75	0.42	0.37	0.39
Frozen base	VGG16	0.82	0.42	0.55	0.47
	ResNet50	0.84	0.53	0.59	0.56
	Xception	0.84	0.43	0.61	0.50
Fine-tuning	VGG16	0.88	0.68	0.71	0.70
	ResNet50	0.87	0.51	0.76	0.61
Ensemble	all pre-trained	0.86	0.79	0.62	0.70

## Data Availability

The data presented in this study are available on request from the corresponding author.
